# Supplementary figures and images for: Can Retinal Ganglion Cell Dipoles Seed Iso-Orientation Domains in the Visual Cortex?
Source: PLoS One. 2014 Jan 24;9(1):e86139. doi: 10.1371/journal.pone.0086139 (PMC3901677; doi:10.1371/journal.pone.0086139)

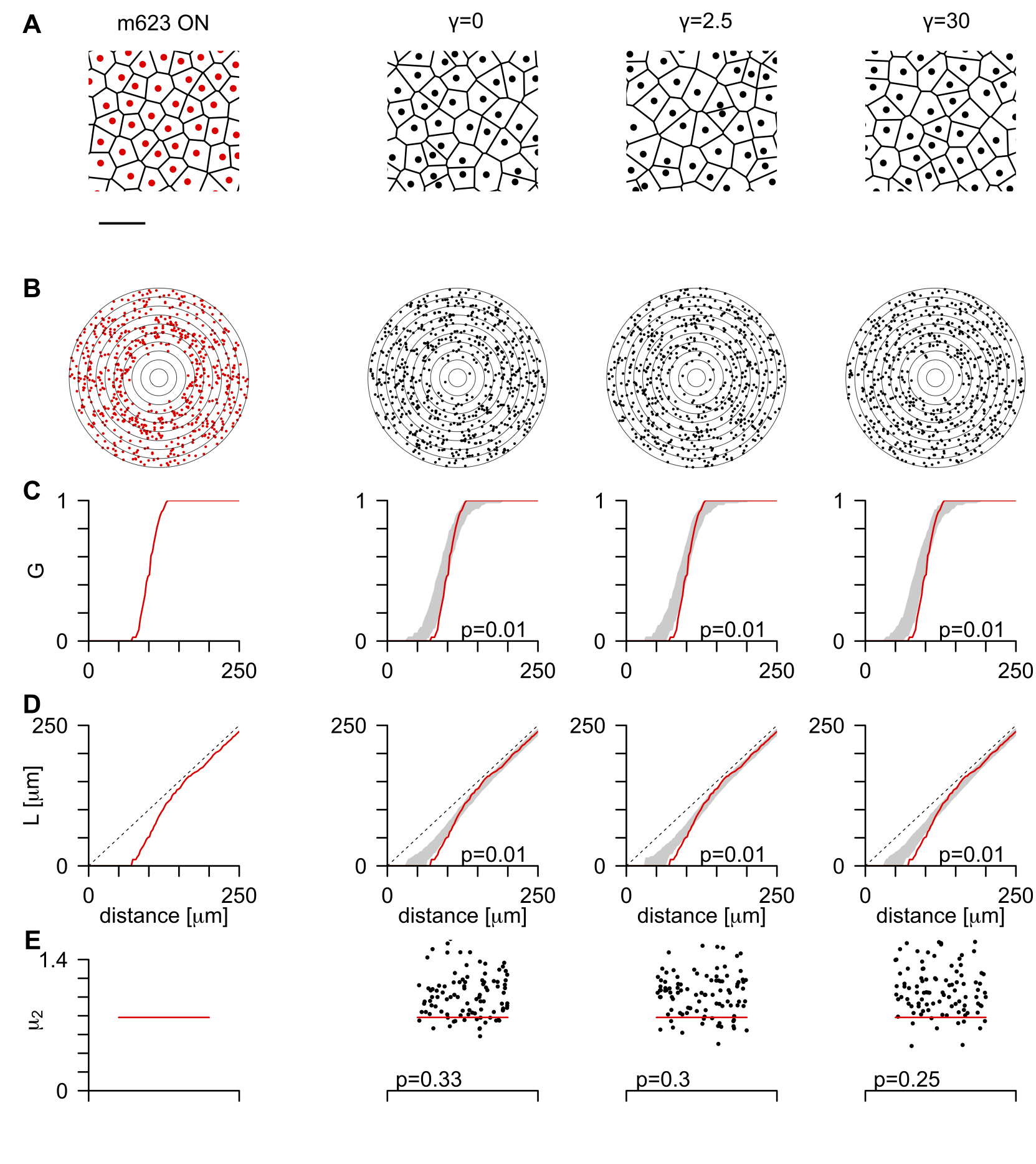

Supplement: Figure S1 — Spatial properties of ON cell positions in mPIPP mosaics are independent of and in agreement with experimental data. Same as Fig. 6, but for ON cells in RGC mosaic m623. (TIF) [file pone.0086139.s001.tif]
