# Supplementary material for: Can Retinal Ganglion Cell Dipoles Seed Iso-Orientation Domains in the Visual Cortex?
Source: PLoS One. 2014 Jan 24;9(1):e86139. doi: 10.1371/journal.pone.0086139 (PMC3901677; doi:10.1371/journal.pone.0086139)
Supplement: Table S1 — Model parameters for PIPP and mPIPP simulations used in the current study. Parameters are chosen such that PIPP mosaics match the local spatial statistics of m623, w81s1 (see [34]) and G09 (see [33]). (PDF) [file pone.0086139.s002.pdf]

Supplementary Table 1

**Mosaic key**	**Parameters**								
	n_{ON}	n_{OFF}	$l_x[\mu m]$	$l_y[\mu m]$	$\varphi_{\mathrm{ON}}[\mu m]$	$\varphi_{\mathrm{OFF}}[\mu m]$	α_{ON}	α_{OFF}	$\delta[\mu m]$
w81S1	65	70	750	991	67.94	66.27	7.81	5.40	18
m623	74	82	1002	1100	112.79	65.46	3.05	8.11	20
G09	89	117	1850	1075	130	125	14.5	13.0	20
